# Current status and future perspectives of low-temperature electrolytes for supercapacitors

**DOI:** 10.1039/d5sc03933a

**Published:** 2025-07-15

**Authors:** Changde Ma, Pratteek Das, Xiaoyu Shi, Feng Zhou, Zhong-Shuai Wu

**Affiliations:** a State Key Laboratory of Catalysis, Dalian Institute of Chemical Physics, Chinese Academy of Sciences 457 Zhongshan Road Dalian 116023 China wuzs@dicp.ac.cn; b Dalian National Laboratory for Clean Energy, Chinese Academy of Sciences 457 Zhongshan Road Dalian 116023 China

## Abstract

Supercapacitors are critical for high-power applications due to their fast charge–discharge capabilities and long lifespans. However, achieving high performance at ultra-low temperatures remains a significant challenge, limiting their use in extreme environments. The electrolyte responsible for ion transport is the key factor governing the low-temperature performance of supercapacitors. In this perspective, we focus on the recent advances in low-temperature electrolytes for supercapacitors. We first introduce the critical physical parameters for evaluating low-temperature electrolytes. Then, we emphasize the key design strategies for low-temperature electrolytes, followed by a detailed discussion of their anti-freezing mechanisms, encompassing aqueous, organic, ionic liquid, and gel-based electrolytes. Additionally, particular emphasis is placed on the theoretical simulation and advanced characterization techniques, given their capability to elucidate the microscopic structure of the electrolyte and provide comprehensive insights into its energy storage mechanism. Finally, we have provided an outlook on the current challenges and future development directions of low-temperature electrolytes, which is expected to offer promising strategies for reliable, high-performance supercapacitors in ultra-low temperature applications.

## Abstract 2

This perspective highlights recent advances in low-temperature electrolytes for supercapacitors, covering key physical parameters, typical design strategies, theoretical simulations and advanced characterization techniques.

## Graphical abstract

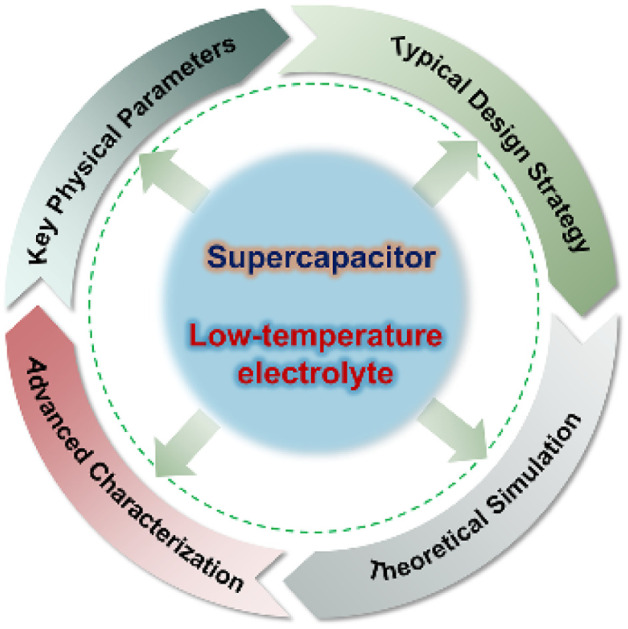

## 1. Introduction

Supercapacitors represent a promising class of energy storage devices, known for their rapid charge/discharge rates and long cycle life, enabling them to be ideal candidates for high-power applications.^1^ Although they cannot replace traditional batteries in terms of energy density, supercapacitors can sustain high charge/discharge rates for more than one million cycles and enable energy recovery in heavy-duty systems.^2–4^ In general, supercapacitors store energy through charge adsorption and desorption or surface pseudocapacitive reactions, avoiding the obvious volume changes in batteries from bulk-phase intercalation reactions, which is the main reason for limited cycle life in batteries (typically hundreds to a few thousand cycles).^5–7^ Due to the high power and long lifespan, supercapacitors have widespread applications in fields such as high-altitude areas, military equipment, polar exploration and aerospace. These applications often require supercapacitors to operate in harsh environments, where temperatures can sometimes drop below −40 °C or even lower.^8^ However, at such temperatures, the viscosity of conventional electrolytes increases significantly, and they may even solidify, resulting in a substantial decrease in conductivity, which can lead to a decline in device performance or even failure. In such situations, supercapacitors require special protective measures, such as external heating or thermally insulating casings, which not only increase the overall weight and design complexity but also raise the total cost.

The electrolyte, responsible for ion transport, is the key factor determining the supercapacitors' low-temperature capabilities. However, conventional electrolytes, with their high freezing points and slow ion transport kinetics, limit the applications of supercapacitors in low-temperature environments. Specifically, at low temperature, the sluggish ion migration in the bulk electrolyte results in an increased impedance; meanwhile, the increase in viscosity triggers severe concentration polarization. Furthermore, there is an obstruction to ion diffusion at the electrode/electrolyte interface, resulting in a significantly increased charge transfer impedance.^9^ These factors result in severe capacitance decay. To overcome these challenges, it is essential to develop electrolytes that not only decrease the freezing point but also exhibit other crucial characteristics, including superior ionic conductivity, low viscosity, and excellent chemical stability at low temperature.

**Fig. 1:**
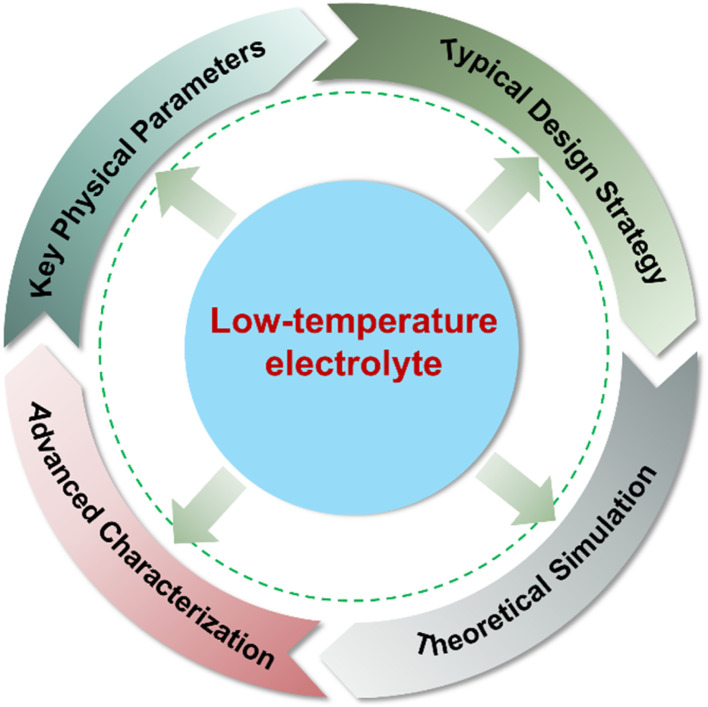
Schematic illustration of the main contents for low-temperature electrolytes of supercapacitors.

In this perspective, we first provide a comprehensive discussion on the intrinsic physical properties of low-temperature electrolytes. Subsequently, we summarize the key strategies reported to address the related challenges, focusing on aqueous, non-aqueous and gel electrolytes. Afterwards, theoretical simulations and advanced characterization techniques are briefly outlined to better analyze the underlying energy storage mechanism at low temperatures (Fig. 1). Finally, strategic research directions for further development are proposed, offering rational guidance and new insight for designing low-temperature electrolytes towards high-performance supercapacitors.

## 2. Key physical parameters

To optimize the performance of electrolytes at low temperatures, a systematic approach and corresponding optimization strategies for various intrinsic physical properties (such as freezing point, viscosity, ionic conductivity, and electrochemical stability window) are essential. Besides, it is crucial not to focus on a single parameter in isolation, but rather to consider the balance between multiple factors, when designing low-temperature electrolytes. For example, adding organic solvents into aqueous electrolytes can effectively lower the freezing point, but may also result in reduced ionic conductivity.^10^ Similarly, while incorporating organic solvents into ionic liquids can reduce the freezing point and viscosity, and increase ionic conductivity, it may also slightly diminish the electrochemical stability window.^11^ In addition, water-in-salt (WIS) electrolytes have been shown to have a wider working voltage and a lower freezing point, but due to their inherently high viscosity, the ionic conductivity is lower. In fact, the solubility of some salts is greatly decreased at lower temperature which results in salt precipitation, leading to the blockage of ion transport paths in separators and electrodes' pores. Therefore, the concentration and composition of the electrolyte should be carefully optimized to provide a trade-off between ionic conductivity and freezing point.^12,13^

To ensure the optimal performance of supercapacitors at low temperatures, the primary consideration is to prevent the electrolyte from freezing. This puts the emphasis on depressing the freezing point to ensure rapid ion movement in electrolyte. For instance, aqueous electrolytes typically have a higher freezing point due to the presence of extensive hydrogen bonding between water molecules. The cryogenic phase transition arises from thermodynamically dominant hydrogen-bond networks that surpass the thermal motion of water molecules at reduced temperatures, inducing spontaneous molecular reorganization into hexagonal crystalline lattices through enthalpy-driven nucleation processes. Therefore, weakening these hydrogen bonds is an effective strategy to extend the operating temperature. In contrast, non-aqueous electrolytes have a lower freezing point, but they also suffer from the issue of significant increase in intermolecular forces, or even freezing at temperatures below −40 °C. For extreme low-temperature applications, it is crucial to carefully design the electrolyte with low freezing point by disrupting the strong interactions between the solvents.

Viscosity (*η*), as a parameter that describes a liquid's resistance to flow, is closely linked to ion transport behavior. Both ionic conductivity and the diffusion coefficient are affected by viscosity. Higher viscosity generally corresponds to lower ionic conductivity and a smaller diffusion coefficient. Additionally, the wetting properties of the separator and electrode materials are also influenced, with high-viscosity electrolytes typically wetting slower than low-viscosity ones.^14^ Therefore, the viscosity of the electrolyte significantly impacts the performance of supercapacitors. The exponential relationship (*η* = *η*_0_e^−*E*_b_/*αK*_B_*T*^) illustrates that even a slight decrease in temperature can cause notable variations in viscosity.^8^ As a result, high-performance electrolytes that work well at room temperature often have subpar performance at low temperatures owing to increased viscosity. Generally, the viscosity of an electrolyte is determined by the type and concentration of the solvent and salt. Compared to pure solvents, mixed solvents are more commonly employed in low-temperature electrolytes due to the introduction of low-viscosity organic solvents.^15^ In addition to the solvent, the salt also affects the electrolyte's viscosity. Generally, the coulombic interactions between cations and anions are stronger than the dipole–dipole interactions between solvent molecules, resulting in an increase in viscosity. Moreover, high salt concentration results in the formation of ion pairs or aggregates, and the presence of these aggregates increases the internal friction of the solution. Meanwhile, the interactions between ions (such as electrostatic forces) are enhanced, inducing an increase in viscosity.^16^

The ionic conductivity (*σ*) of an electrolyte influences the equivalent series resistance of supercapacitors, which in turn affects the rate performance and power density. Ionic conductivity is primarily dependent on the free-moving ions in the solution. As the temperature decreases, ionic conductivity typically decreases gradually at first and then more sharply below the freezing point. This is attributed to ion aggregation or salt precipitation occurring at lower temperatures, which reduces the number of free-moving ions in the solution, thereby affecting the ionic conductivity. Additionally, ionic conductivity is influenced by the concentration of the electrolyte. It generally increases as ion concentration rises. However, at relatively high concentrations, stronger ion interactions may occur and reduces the number of free-moving ions, potentially resulting in a reduction in conductivity. In addition to temperature and ion concentration, the nature of the charge carriers also plays a significant role in determining ionic conductivity. For instance, solvated cations with smaller solvated radii tend to exhibit high ionic conductivity.

The electrochemical stability window defines the voltage range in which the electrolyte remains stable and does not decompose. In general, electrochemical stability window is largely determined by the highest occupied molecular orbital (HOMO) and lowest unoccupied molecular orbital (LUMO) energy levels of solvents.^17^ However, Peljo *et al.* suggested that the concept of HOMO and LUMO, originally derived from studies on the electronic properties of isolated molecules, may not be directly applicable to complex electrolytes.^18^ As a result, the redox reactions of multi-component electrolytes cannot be solely evaluated from the HOMO–LUMO energy gap. It has been suggested that the electrochemical stability of electrolytes should be more accurately described by considering oxidation and reduction potentials instead. At low temperatures, the electrochemical stability window of electrolytes can be significantly affected due to changes in the electrolyte's physical and chemical properties. In some cases, at lower temperatures, solvent decomposition is inhibited by slowing down side reactions at the interface, thus elevating the electrolyte's electrochemical stability windows.^19^

**Fig. 2:**
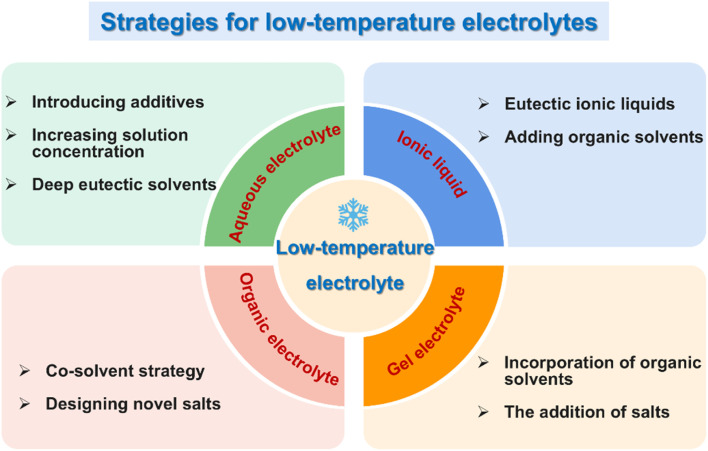
The key design strategies for developing different low-temperature electrolytes for supercapacitors.

In short, the physical parameters of electrolytes are crucial for the design and performance improvement of low-temperature supercapacitors. To design high-performance electrolytes, it is essential to consider not only individual parameters but also the balance between various factors. Based on the above, diverse strategies have been employed to design various types of low-temperature electrolytes, including aqueous, organic, ionic liquid, and gel electrolytes. A comprehensive analysis of each strategy, along with its corresponding anti-freezing mechanisms, will be systematically discussed in the subsequent sections (Fig. 2).

## 3. Key design strategies

### 3.1. Aqueous electrolytes

Aqueous electrolytes have garnered widespread interest in supercapacitors due to their superior ionic conductivity, non-flammability, low cost, and environmental benefits.^20–22^ Beyond traditional supercapacitors, zinc-ion hybrid supercapacitors (ZHSs) have emerged as a class of aqueous energy storage devices that integrate high power density of supercapacitors with high energy density of batteries.^23,24^ Typically, ZHSs consist of a capacitive carbon-based cathode and a battery-type zinc anode, operating through a synergistic charge storage mechanism that combines rapid surface adsorption/desorption at the cathode with reversible zinc plating/stripping at the anode. However, given the relatively high freezing point of water, aqueous electrolytes tend to freeze within 0 to −20 °C. To enable supercapacitors to operate under colder conditions, several approaches have been introduced to decrease the freezing point and enhance ionic conductivity, with the most effective approaches including introducing anti-freezing additives, increasing electrolyte concentration, and utilizing deep eutectic solvents (DESs). The typical low-temperature aqueous electrolytes for supercapacitors are summarized in Table 1.

**Table 1:** A list of typical low-temperature aqueous electrolytes for supercapacitors^a^

Electrolyte	Concentration	Ionic conductivity [mS cm^−1^]	Specific capacitance/capacity	Operating temperature [^°^C]	Electrode material (device type)	Voltage [V]	Ref.
CaCl_2_ in H_2_O	3.8 m	10.1 (−50 °C)	59.3 F g^−1^ (0.5 A g^−1^, 25 °C)	−50	AC (SCs)	1.8	25
Zn(OTf)_2_ in H_2_O/ACN	2 M	54.2 (25 °C)	64 mF cm^−2^ (500 mV s^−1^, −40 °C)	−40	MXene@CC//Zn (ZHSs)	1.6	26
Ca(ClO_4_)_2_ in H_2_O/ACN	4.2 m	1.5 (−50 °C)	46 F g^−1^ (1 A g^−1^, −50 °C)	−50	AC (SCs)	2.3	22
CaCl_2_ in EG/H_2_O	3.86 m	0.69 (−50 °C)	18.4 mF cm^−2^ (0.1 mA cm^−2^, 20 °C)	−40	AC (SCs)	1.6	27
LiTFSI in H_2_O	5 m	50 (RT)	22 F g^−1^ (10 A g^−1^, 15 °C)	−30	AC (SCs)	2	13
LiTFSI in ACN/H_2_O	5 m	17.4 (25 °C)	8.6 F g^−1^ (10 A g^−1^, −30 °C)	−30	AC (SCs)	2.2	28
LiNO_3_ in H_2_O/1,3-propylene glycol	3.65 m	67.5 (25 °C)	20.6 F g^−1^ (5 mV s^−1^, −40 °C)	−40	AC (SCs)	2.0	20
3NaClO_4_–2CO(NH_2_)_2_/H_2_O	—	135 (25 °C)	22.6 F g^−1^ (20 mV s^−1^, −30 °C)	−40	AC (SCs)	2.25	29
		5.3 (−50 °C)					
CsAc in H_2_O	10 m	0.8 (−80 °C)	200 mF cm^−2^ (0.1 mA cm^−2^, −95 °C)	−95	AC (SCs)	0.8	30
H_2_SO_4_–ZnCl_2_ acid-salt hybrid electrolyte	0.2–5.4 M	6.97 (−60 °C)	39.8 mA h g^−1^ (1 A g^−1^, −80 °C)	−80	VHCF//α-MoO_3_ (hybrid capacitors)	1.5	31

a VHCF: Prussian blue analog, SCs: supercapacitors, AC: activated carbon.

**Fig. 3:**
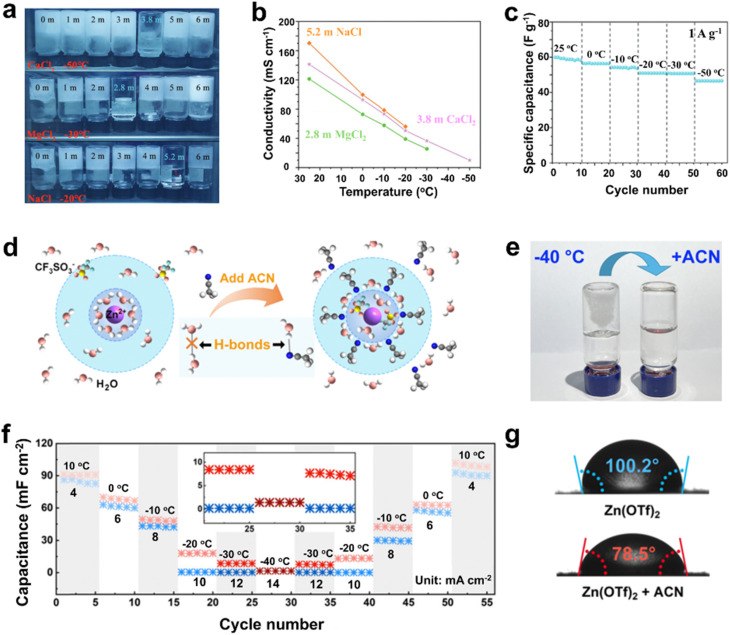
Low-temperature aqueous electrolytes engineered by utilizing inorganic salts (*e.g.*, CaCl_2_) or organic solvents (*e.g.*, ACN) as antifreeze additives. (a) Digital photos of the BREs and control electrolytes. (b) Variation in ionic conductivity of the BREs with temperature. (c) Change in capacitance of the supercapacitor at 1 A g^−1^ with a gradual decrease in temperature from 25 °C to −50 °C. Reproduced with permission.^25^ Copyright 2023, Wiley-VCH. (d) Schematic illustration of Zn^2+^ solvent sheath in Zn(OTf)_2_ electrolyte (left) and Zn(OTf)_2_ + ACN electrolyte (right). (e) Photograph of the two types of electrolytes at −40 °C. (f) The capacitance of supercapacitors using the two types of electrolytes at varying temperatures, with pink representing the electrolyte containing ACN. (g) The contact angle between the Zn anode and different electrolytes. Reproduced with permission.^26^ Copyright 2025, Royal Society of Chemistry.

Anti-freezing additives are typically selected for their capacity to establish strong coordination or hydrogen bonds with water molecules, as well as their high solubility in water. The most-commonly reported anti-freezing additives include inorganic salts and certain organic co-solvents, like ethylene glycol (EG), acetonitrile (ACN), and glycerol.^10^ For example, considerable research has focused on incorporating calcium chloride (CaCl_2_) into aqueous electrolytes to improve their antifreeze properties.^32,33^ The intense interaction between CaCl_2_ and water molecules disrupts the original hydrogen bonding framework, leading to a lower freezing point and an expanded operating voltage. As a typical example, You and co-workers developed a CaCl_2_-based brine refrigerant electrolyte (BRE) for supercapacitors, featuring a significantly reduced freezing point (−55 °C) and sustaining high ionic conductivity (10.1 mS cm^−1^) even at −50 °C. This electrolyte enables the supercapacitors to retain 80% of their room-temperature capacitance at −50 °C while demonstrating superior cycle life and outstanding capacitance retention (Fig. 3a–c).^25^ Furthermore, organic co-solvents with strong water-coordinating capabilities or low freezing points have been introduced into aqueous electrolytes to disrupt the hydrogen bonding, effectively suppressing the freezing of the aqueous solution. For example, Liu *et al.* employed ACN as a low-temperature co-solvent to modify the aqueous electrolyte. The ACN additive disrupted hydrogen bonding between water molecules and restructured the Zn^2+^ solvation sheath, thereby reducing ion transfer resistance and enabling highly reversible Zn/Zn^2+^ redox chemistry. Consequently, the Zn-ion hybrid supercapacitor delivered an area-specific capacitance of 64.0 mF cm^−2^ at 500 mV s^−1^ (−40 °C) and exhibited long-term cyclability (Fig. 3d–g).^26^ In addition, several studies have explored the use of both inorganic salts and organic solvents as additives to create low-temperature electrolytes. As exemplified, Yang *et al.* proposed a cost-efficient hybrid electrolyte for low-temperature micro-supercapacitors (MSCs) by incorporating CaCl_2_ and EG. The addition of CaCl_2_ decreases the quantity of water molecules involved in strong hydrogen bonding, while EG further minimizes the water molecules in the primary solvation shell. This combination yields an electrolyte that exhibits a high voltage stability of 3.5 V and an extremely low freezing point of −120 °C. Consequently, the MSCs retain 62% capacitance at −40 °C compared to their room-temperature performance.^27^

**Fig. 4:**
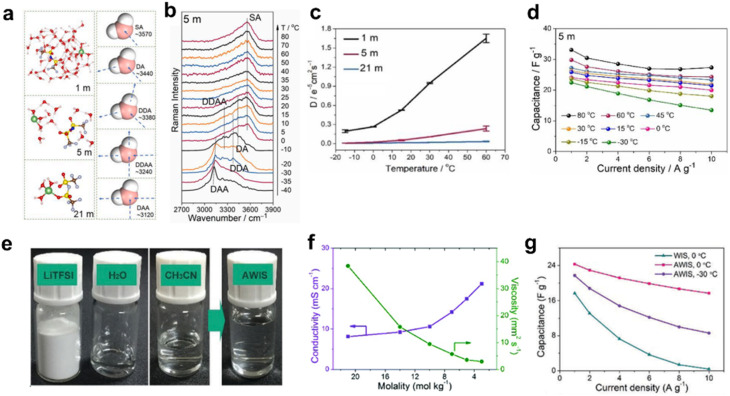
Design strategy for low-temperature aqueous electrolytes based on WIS electrolytes. (a) The left panel illustrates structural models of LiTFSI/H_2_O systems at three distinct concentrations, while the right panel depicts the correlation between water's hydrogen-bonding configurations and their corresponding Raman spectral features. (b) Raman spectral analysis of the O–H stretching region in 5 m LiTFSI/H_2_O systems across varying temperatures. (c) Diffusion coefficients of Li^+^ ions at various temperatures. (d) Evaluation of temperature-dependent specific capacitance in supercapacitors employing 5 m LiTFSI aqueous electrolytes. Reproduced with permission.^13^ Copyright 2023, Elsevier. (e) Images illustrating the preparation of AWIS electrolytes with various concentrations. (f) Influence of electrolyte concentration on ionic conductivity and viscosity. (g) Comparison of the specific capacitance at different current densities. Reproduced with permission.^28^ Copyright 2018, Royal Society of Chemistry.

The WIS electrolytes have experienced rapid development in electrochemical energy storage devices, following their initial proposal by Suo *et al.* in 2015.^34^ Recently, WIS electrolytes have demonstrated great potential for use in supercapacitors, owing to their wide electrochemical stability window and excellent ion transport properties.^16,21,35^ Generally, the added salt forms hydrated ions with free water molecules, decreasing the number of hydrogen bonds and thereby lowering the freezing point. By exploiting this property, high-concentration electrolytes have been developed to function as low-temperature electrolytes for supercapacitors. For instance, Zhang and colleagues found that the 5 m LiTFSI/H_2_O electrolyte exhibits excellent low-temperature behaviors even at −30 °C. They also explored the temperature-related electrochemical properties of electrolytes with different concentrations, analyzing their intermolecular interactions and solvation structures using molecular dynamics simulations and Raman spectroscopy (Fig. 4a–d).^13^ However, it is noted that as the salt concentration increases to a higher level, salt crystallization is likely to occur at low temperatures, leading to performance decline or potential failure of supercapacitors. This issue can be effectively mitigated by incorporating organic solvents into the high-concentration electrolyte. As a representative example, Dou *et al.* developed a co-solvent-in-salt system by introducing ACN into a 21 m LiTFSI/H_2_O electrolyte, creating an ACN/WIS electrolyte (AWIS). This work demonstrates that combining highly concentrated aqueous electrolytes with organic solvents is a promising strategy for high-performance supercapacitors with both a high voltage and wide temperature range (Fig. 4e–g).^28^

**Fig. 5:**
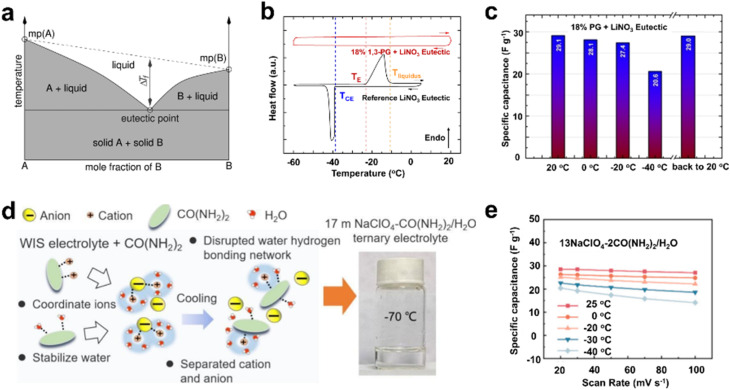
DES strategy for low-temperature electrolyte design. (a) Schematic depiction of the eutectic point in a binary phase diagram. Reproduced with permission.^36^ Copyright 2014, American Chemical Society. (b) Thermal analysis curves of the LiNO_3_-based eutectic and the eutectic containing 18% 1,3-propylene glycol. (c) Capacitance retention at different temperatures. Reproduced with permission.^20^ Copyright 2023, Wiley-VCH. (d) Schematic depiction of the proposed ternary aqueous electrolyte. (e) Rate capability of the supercapacitor utilizing the 13NaClO_4_–2CO(NH_2_)_2_/H_2_O electrolyte at various temperatures. Reproduced with permission.^29^ Copyright 2024, Elsevier.

DESs are typically binary or multi-component mixtures composed of a hydrogen bond acceptor and a hydrogen bond donor, with the eutectic point significantly lower than that of its individual components (Fig. 5a).^36^ Certain aqueous salt hydrate systems, consisting of mixtures of ice and partially dissolved salt hydrates under low-temperature conditions, can also be considered as DESs. The eutectic point of such aqueous solutions can be determined using a solid–liquid phase diagram. Additionally, thermodynamic simulations can be employed to generate phase diagrams for such electrolytes, aiding in the identification of the optimal salt-to-water proportion needed to attain the target freezing point.^37^ At the “eutectic concentration”, the mixture shows the lowest freezing point.^38^ In recent years, DESs have garnered significant attention as promising electrolytes for supercapacitors operating at low temperatures. For instance, Ah-Lung *et al.* proposed an environmentally friendly and cost-effective ternary deep eutectic electrolyte system composed of LiNO_3_, water, and 1,3-propylene glycol, aiming to design an electrolyte with a broad liquid phase range. When used in supercapacitors, this electrolyte enabled the device to achieve a minimal capacitance loss of only 6% at −20 °C and 29% at −40 °C (Fig. 5b and c).^20^ As another typical example, Bo *et al.* put forward a ternary interaction approach involving cations, water, and urea to create electrolytes suitable for low-temperature applications, demonstrating aqueous electrolytes with an exceptionally depressed freezing point and superior ionic conductivity. The optimized ternary aqueous electrolyte achieves an extremely low freezing point of −70 °C and exhibits a remarkable ionic conductivity of 5.3 mS cm^−1^ at −50 °C. Even at −40 °C, the supercapacitors employing the 13NaClO_4_–2CO(NH_2_)_2_/H_2_O electrolyte exhibit approximately 70% capacitance retention with the scan rate increasing from 20 to 100 mV s^−1^, significantly surpassing that of supercapacitors using a 17 m NaClO_4_ WIS electrolyte (Fig. 5d and e).^29^

**Fig. 6:**
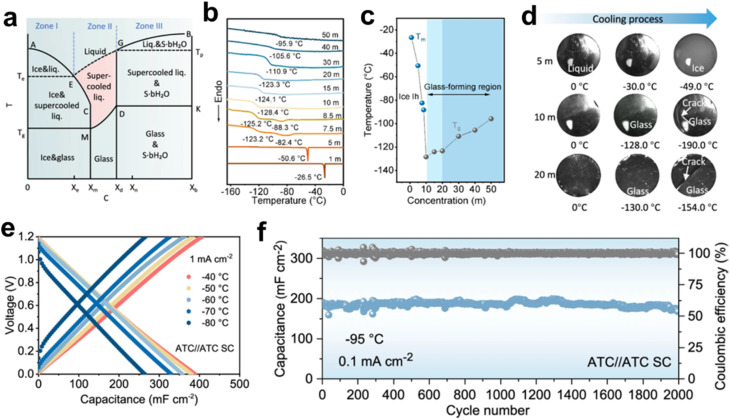
Entropy-engineered electrolyte systems for low-temperature supercapacitors. (a) Schematic representation of the phase behavior in a non-equilibrium binary system comprising water and salt. (b) DSC measurements of CsAc aqueous solutions at varying concentrations. (c) Dependence of primary phase transition temperatures on CsAc concentration. (d) *In-situ* optical microscopy observations of electrolytes during the cooling process. (e) Charge–discharge profiles at different temperatures. (f) Long-term cyclability observed at −95 °C. Reproduced with permission.^30^ Copyright 2024, Springer Nature.

**Fig. 7:**
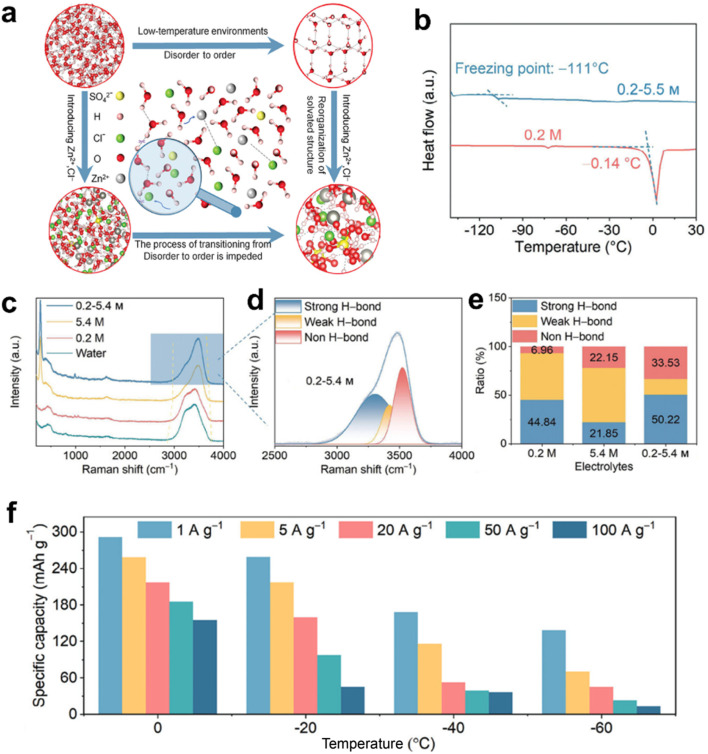
Acid-salt hybrid electrolytes for low-temperature supercapacitors. (a) Diagram of the structure evolutions for designing low-temperature electrolytes. (b) The DSC curves of different electrolytes. (c) The Raman spectra of different electrolytes. (d) The fitted O–H stretching vibration in 0.2–5.4 m electrolyte. (e) The proportion of strong, weak, and non H-bonds in different solutions. (f) The specific discharge capacity at various current densities and temperatures. Reproduced with permission.^31^ Copyright 2024, Wiley-VCH.

Extending the operational temperature range of aqueous electrolytes has emerged as a significant research focus, with recent advances unveiling novel mechanism insights. For example, entropy regulation of specific electrolyte systems may offer a novel strategy for depressing the freezing point of aqueous solutions. In this context, Qiu *et al.* proposed the concept of entropy-driven glass-forming liquids (EDGFLs) as an innovative paradigm for designing freeze-resistant electrolytes. Through precise modulation of local structural ordering, the glass-transition temperature (*T*_g_) can be systematically engineered to suppress energy-dominated ice crystallization while facilitating entropy-driven glass transition. This behavior can be fundamentally understood through the competitive interplay between ion pair correlation entropy and water's tetrahedral entropy. Moreover, experimental validation is achieved through differential scanning calorimetry (DSC) and *in situ* optical microscopy. The resulting EDGFL system, exhibiting an ultralow *T*_g_ of −128 °C, enables supercapacitors to facilitate superior capacitance retention under cryogenic conditions while sustaining operational stability down to −95 °C (Fig. 6).^30^ Furthermore, acid-salt hybrid electrolytes featuring stable anion–cation-H_2_O solvation structures facilitate unconventional proton conduction at cryogenic temperatures. A representative study by Cui *et al.* demonstrated that such hybrid electrolytes can effectively reorganize water network channels to promote efficient ion transport, enabling rapid ionic conductivity and superior rate performance at low temperatures. It is revealed from Raman spectroscopy that the hydrogen bond network structure between water molecules is disrupted by Cl^−^ and Zn^2+^, leading to a depression of the freezing point. While such disruption mitigates solvent crystallization at low temperatures, it is important to note that the strong yet localized hydrogen bonding, particularly between the ions and solvent molecules, can persist within the system. These localized interactions may facilitate the formation of stable solvation structures and continuous ion conduction pathways, which are beneficial for maintaining efficient ion transport under cryogenic conditions. As a result, a reversible capacity of 151 mA h g^−1^ (46.6% of room-temperature capacity) can be retained at 1 A g^−1^ even at −60 °C, highlighting the electrolyte's superior low-temperature adaptability (Fig. 7).^31^

### 3.2. Organic electrolytes

**Fig. 8:**
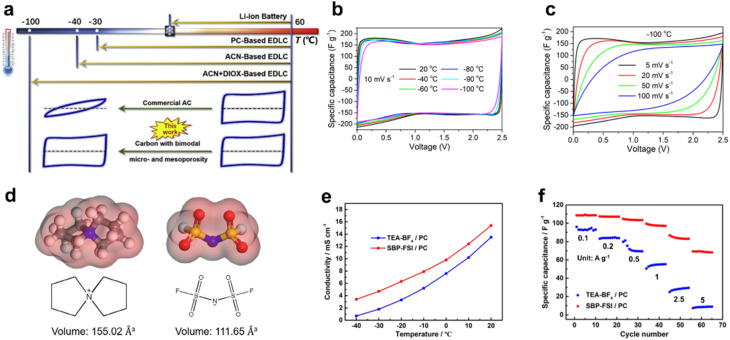
Design of a low-temperature organic electrolyte *via* co-solvent formulation or novel salt engineering. (a) Schematic depiction of SC behavior at various temperatures. (b) CV curves of supercapacitors from 20 to −100 °C (c) CV curves measured at −100 °C with different scan rates. Reproduced with permission.^15^ Copyright 2019, Elsevier. (d) Molecular structures of the electrolyte components. (e) Ionic conductivities of SBP-FSI/PC based electrolytes and TEA-BF_4_/PC commercial electrolytes at different temperatures. (f) The rate capability of supercapacitors based on these two electrolytes. Reproduced with permission.^40^ Copyright 2019, Elsevier.

Compared to aqueous electrolytes, organic electrolytes contribute to lower operating temperatures and a wider electrochemical stability window. To achieve high ionic conductivity, commercial electrolytes typically utilize organic solvents with high dielectric constants, such as propylene carbonate or ACN, which facilitate the complete dissociation of salts like tetraethylammonium tetrafluoroborate (TEABF_4_). However, high dielectric constants are usually associated with high freezing points due to the strong dipole–dipole interactions between solvent molecules, which leads to a rapid decline in ionic conductivity at low temperatures. When the temperature drops to below −40 °C, even the organic electrolyte does not freeze like aqueous electrolytes, but its ionic conductivity still decreases significantly owing to the increase in viscosity. Therefore, an effective strategy to enhance ion transport and improve low-temperature performance is to introduce a co-solvent with a lower freezing point and a reduced dielectric constant. For instance, Xu *et al.* proposed a solvent mixture of acetonitrile (ACN) and 1,3-dioxolane that enables supercapacitor operation at temperatures down to −100 °C, thereby outperforming traditional ACN-based systems. This innovative combination leads to a record gravimetric capacitance of 164 F g^−1^ at −100 °C, which is nearly identical to the capacitance measured at room temperature (Fig. 8a–c).^15^ Besides, high-entropy electrolytes have also been developed to enhance the performance of supercapacitors.^39^ By incorporating multiple solvents, the solvation structure of the electrolyte can be tailored, thereby increasing the system's configurational entropy. This strategy can effectively suppress the freezing point and facilitate faster desolvation kinetics to improve low-temperature electrochemical performance. As a result, the rate capability of the electrolyte is significantly enhanced.

Apart from the solvent, novel salts with excellent solubility, high ionic conductivity, and superior electrochemical stability, such as spiro-(1,1′)-bipyrrolidinium bis(fluorosulfonyl)imide (SBP-FSI), have been progressively developed for organic electrolytes.^40–42^ Electrochemical characteristics of the electrolyte are largely determined by the specific types of ions present in the salt. In comparison to the commercially available electrolyte salt (TEABF_4_), SBP-FSI exhibits superior performance at low temperatures. Moreover, SBP^+^ also has a smaller molecular volume and a shorter distance between the ion center and electrode surface, thereby contributing to a higher theoretical specific capacitance. For instance, Zhang *et al.* demonstrated that the combination of SBP-FSI and propylene carbonate (PC) displays greater ionic conductivity at low temperatures for supercapacitors compared to TEA-BF_4_/PC. As a result, SBP-FSI/PC based supercapacitors showed excellent rate capabilities at low temperature, exhibiting 64.8% capacitance retention *versus* TEA-BF4/PC's near-complete performance collapse (90% reduction) under 5 A g^−1^ at −40 °C (Fig. 8d–f).^40^

### 3.3. Ionic liquid electrolytes

**Fig. 9:**
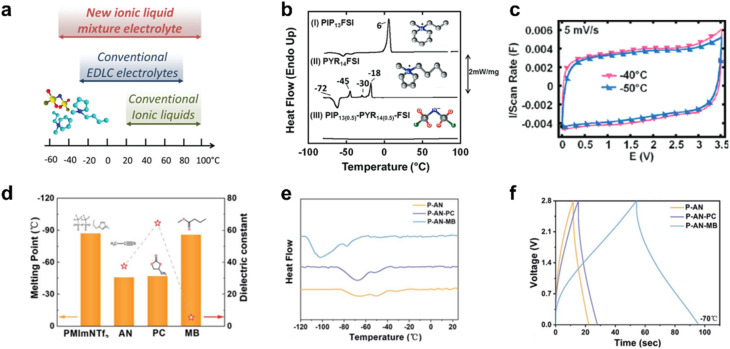
Low-temperature ionic liquid electrolytes developed through eutectic composition or organic co-solvent strategies. (a) Employing a eutectic ionic liquid mixture enables supercapacitor operation at temperatures as low as −50 °C. (b) DSC thermograms of (I) PIP_13_FSI, (II) PYR_14_FSI and (III) (PIP_13_FSI)_0.5_(PYR_14_FSI)_0.5_ mixture, along with their molecular structures. (c) CV curves measured in the ionic liquid mixture electrolyte at low temperatures. Reproduced with permission.^46^ Copyright 2011, American Chemical Society. (d) Physical characteristics of the electrolyte components. (e) DSC curves of different electrolytes. (f) GCD profiles of P-AN, P-AN-PC, and P-AN-MB (AN: ACN) electrolyte-based supercapacitor at −70 °C. Reproduced with permission.^47^ Copyright 2022, Royal Society of Chemistry.

Ionic liquid electrolytes are widely used in supercapacitors owing to their exceptional thermal stability, wide electrochemical stability window, and low flammability, offering both high energy density and enhanced safety.^11,43–45^ However, conventional single-component ionic liquid electrolytes typically exhibit high viscosity and may even freeze at low temperatures, resulting in significantly lower ionic conductivity compared to organic electrolytes under such conditions. To address these issues, several strategies have been adopted to expand the operating temperature. One promising approach is the use of eutectic ionic liquids, which are formed by mixing two or more ionic liquids, preventing the ordered arrangement and crystallization of the individual ionic liquids. Eutectic ionic liquids generally exhibit lower viscosity and higher ionic conductivity under low-temperature conditions.^26^ For instance, Lin *et al.* proposed a binary ionic liquid electrolyte characterized by a significantly depressed freezing point of −80 °C. The supercapacitors based on this electrolyte offer exceptional electrochemical performance even at −50 °C (Fig. 9a–c).^46^

Furthermore, incorporating low-freezing-point, low-viscosity organic solvents into ionic liquids presents an effective approach to lowering the freezing point and enhancing ionic conductivity. This enhancement in ionic conductivity results from reduced ion association in ionic liquid-based electrolytes upon solvation by added solvents, which lowers the viscosity and increases ionic mobility. Ideally, organic solvents should be selected to disrupt ion pairing in ionic liquids while minimizing the formation of strongly solvated ion species, thus ensuring high ion mobility. For example, Tang *et al.* demonstrated that incorporating mixed solvents, such as ACN and methyl butyrate (MB), into ionic liquids significantly enhances their low-temperature performance. By combining spectroscopic characterization with theoretical calculations, it was shown that the unique solvation interactions between the ionic liquid and solvents significantly reduce the electrostatic forces between the cations and anions as well as the hydrogen bonding between them (Fig. 9d–f).^47^ However, it is important to note that while adding organic solvents into ionic liquids is an efficient strategy for reducing the freezing point, it can also narrow the electrochemical stability window simultaneously. Therefore, the balance between the operating temperature and working voltage needs to be considered. Representative low-temperature organic and ionic liquid electrolytes are summarized in Table 2.

**Table 2:** A list of typical low-temperature organic and ionic liquid electrolytes for supercapacitors

	Electrolyte	Concentration	Ionic conductivity [mS cm^−1^]	Specific capacitance	Operating temperature [°C]	Electrode material (device type)	Voltage [V]	Ref.
Organic electrolytes	MeEt_3_NBF_4_ in ACN/DIOX.	0.5 M	18.5 (20 °C)	164 F g^−1^ (5 mV s^−1^, −100 °C)	−100	AC (SCs)	2.5	15
	SBP-FSI in PC	1 M	17.4 (20 °C)	70 F g^−1^ (5 A g^−1^, −40 °C)	−40	AC (SCs)	2.7	40
	EMI-TFSI in multiple organic solvents	1 M	3.9 (−50 °C)	39.1 F g^−1^ (5 A g^−1^, RT)	−50	AC (SCs)	3	39
	SBPBF_4_ in ACN/MF	0.3 M	—	157 F g^−1^ (100 mV s^−1^, −100 °C)	−100	AC (SCs)	2.5	48
	[EMIM][BF_4_] in ACN/MA	1 M	—	45 F g^−1^ (0.5 A g^−1^, RT)	−100	AC (SCs)	3.5	19
Ionic liquid electrolytes	(PIP13FSI)0.5(PYR14FSI)0.5	1 : 1	4.9 (20 °C)	—	−50	CNTs (SCs)	3.7	46
	[Pyrr][NO_3_] [Pyrr][TFSI]	0.72 : 1	1 (−10 °C)	209 F g^−1^ (5 mV s^−1^, 25 °C)	−60	AC (SCs)	2.5	49
	PMImNTf_2_ in ACN/MB	1 : 1 : 1	2.46 (−70 °C)	18.6 F g^−1^ (0.5 A g^−1^, −50 °C)	−70	AC, (SCs)	2.8	47

### 3.4. Gel electrolytes

Gel electrolytes are semi-solid electrolytes that help prevent electrolyte leakage in supercapacitors, offering enhanced safety. In addition, they also possess desirable properties, including high ion conductivity, stretchability, flexibility, and self-healing capabilities, which have garnered significant interest. Hydrogels are primary gel electrolytes used in supercapacitors. In these hydrogels, polymer chains interact with water molecules through hydrogen bonds, disrupting the hydrogen bonding network and suppressing ice crystal formation. However, hydrogel electrolytes still tend to freeze as the external temperature drops below −20 °C, which impedes ion migration and causes significant capacitance loss in supercapacitors.^50^ Recently, hydrogels with anti-freezing properties have gained significant attention.^51^ Several approaches have been proposed to lower the freezing points.^52–55^ In brief, the typical low-temperature gel electrolytes for supercapacitors are summarized in Table 3.

**Table 3:** A list of typical low-temperature gel electrolytes for supercapacitors^a^

Electrolyte	Conductivity [mS cm^−1^]	Specific capacitance/capacity	Operating temperature [°C]	Electrode material (applied device)	Voltage [V]	Ref.
LiOTf in PVA/NMP	1.34 (−25 °C)	25.4 mF cm^−2^ (1 mA cm^−2^, 25 °C)	−40	CNT (SCs)	2.5	56
	10.65 (25 °C)					
NaAc in hydrogel	—	205.9 F g^−1^ (0.2 A g^−1^, 20 °C)	−40	AC (SCs)	2	57
LiBr in poly (ionic liquid) hydrogel	26 (25 °C)	163 mF cm^−2^ (1 mA cm^−2^, −20 °C)	−20	AC (SCs)	1	50
Lignin based hydrogel	96 (RT)	150 F g^−1^ (1 A g^−1^, −30 °C)	−30	AC (SCs)	1	58
H_2_SO_4_ in zwitterionic hydrogel	1.51 (−50 °C)	62.0 F g^−1^ (62.5 mA g^−1^, −50 °C)	−50	AC (SCs)	1	59
ZnSO_4_ in hydrogel	7.68 (25 °C)	82.14 F g^−1^ (0.5 A g^−1^, 25 °C)	−20	AC (SCs)	1.4	60
CuSO_4_ in PVA	0.179 (−15 °C)	72.62 F g^−1^ (100 mA g^−1^, 25 °C)	−80	Graphene (SCs)	1	61
ZnSO_4_ in hydrogel electrolyte	24.3 (−20 °C)	117.5 mA h g^−1^ (0.25 A g^−1^, −20 °C)	−20	AC//Zn (ZHSs)	1.8	62
Zn(OTf)_2_ in PVA gel	—	49.1 mA h g^−1^ (5 A g^−1^, 25 °C)	−30	MXene//Zn (ZHSs)	1.2	63

a NMP: *N*-methylpyrrolidone.

**Fig. 10:**
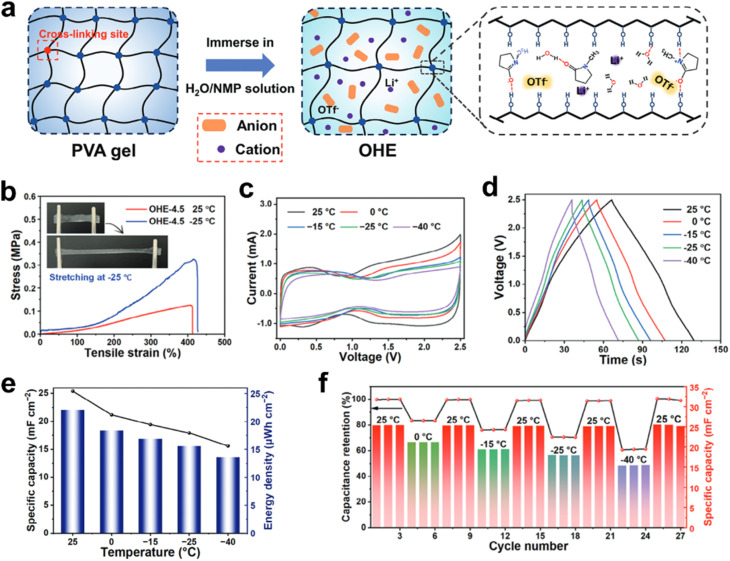
Anti-freezing hydrogel electrolytes designed by introducing organic solvents. (a) Preparation process of the gel electrolyte. (b) Tensile stress–strain curves at 25 and −25 °C of the gel electrolyte. (c) CV curves and (d) GCD profiles of supercapacitors from 25 to −40 °C. (e) Specific capacitance and energy density of supercapacitors at different temperatures. (f) Continuous reversible changes of capacitance retention of supercapacitors under various temperature conditions. Reproduced with permission.^56^ Copyright 2025, Wiley-VCH.

Designing an anti-freezing hydrogel electrolyte involves optimizing several factors to ensure that the hydrogel not only retains its ionic conductivity at low temperatures but also its mechanical stability. In general, the strong hydrogen bonding between water molecules is a major challenge in developing anti-freezing hydrogel electrolytes. To address this, organic solvents are often incorporated into hydrogel electrolytes to disrupt the hydrogen-bonding network of water molecules, effectively suppressing the freezing behavior of the hydrogel electrolyte. For instance, Wang *et al.* proposed the development of anti-freezing hydrogel electrolytes with enhanced water deactivation by incorporating *N*-methylpyrrolidone solvent. As a result, the flexible supercapacitors exhibit nearly rectangular CV curves and symmetrical triangular GCD profiles within the temperature range of −40 to 0 °C. The flexible supercapacitors deliver a record areal energy density of >13.55 μ Wh cm^−2^ with voltage outputs exceeding 2.5 V across −40 to 25 °C. The environmental temperature fluctuation test indicates that the device exhibits excellent environmental adaptability (Fig. 10).^56^ While incorporating organic solvents effectively lowers the freezing point, the mechanical strength of hydrogels remains unsatisfactory owing to the insufficient interactions between polymer chains.^64^ To address this issue, a solution can be found by designing and synthesizing dual-crosslinked hydrogels incorporating ethylene glycol-functionalized groups.^65^

**Fig. 11:**
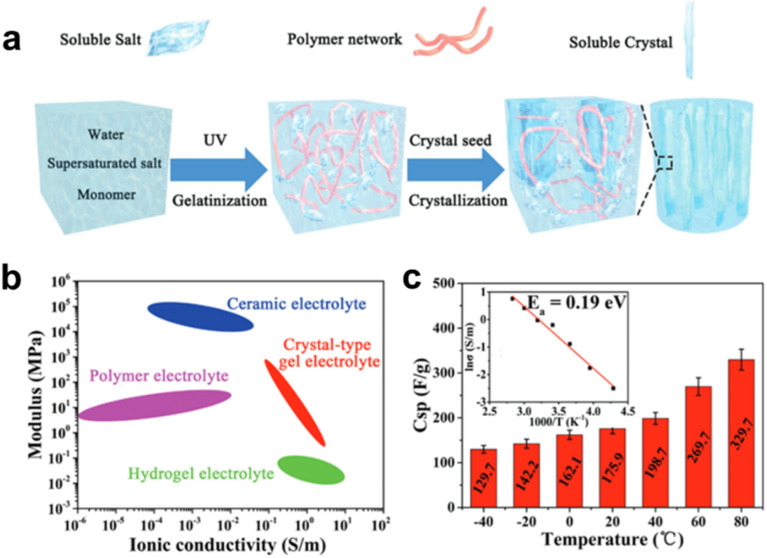
Inorganic salt-modified hydrogel electrolytes enabling low-temperature operation of the supercapacitor. (a) *In situ* crystallization within a hydrogel matrix for the synthesis of crystalline composite gels. (b) Mechanical modulus and ionic conductivity of various non-liquid electrolytes. (c) Specific capacitance of gel electrolyte-based supercapacitors at various temperatures from −40 °C to 80 °C. Reproduced with permission.^57^ Copyright 2019, Wiley-VCH.

The addition of salts can also significantly inhibit the freezing behavior of hydrogel electrolytes.^57,61,66–68^ In general, introducing salt into the system disrupts both inter- and intra-chain hydrogen bonds within the polymer network, thereby promoting stronger interactions between polymer chains and water molecules. However, as the salt concentration continues to increase, these beneficial interactions diminish due to excessive disruption of hydrogen bonding, resulting in salt precipitation. Thus, optimizing the salt concentration is crucial for preparing hydrogel electrolytes with optimal performance. For instance, Wei *et al.* developed a high-performance, processable crystalline gel electrolyte by using the dissolution–crystallization transition of sodium acetate (NaAc) in the hydrogel. The addition of NaAc crystals increases operating voltage (2.0 V) and the modulus (474.24 MPa) compared to the hydrogel without crystals. The resulting supercapacitors perform reliably in extremely low-temperature environments due to its phase-transition ability. Even at −40 °C, the specific capacitance can be maintained at 73.7% of the value at 20 °C (Fig. 11).^57^

**Fig. 12:**
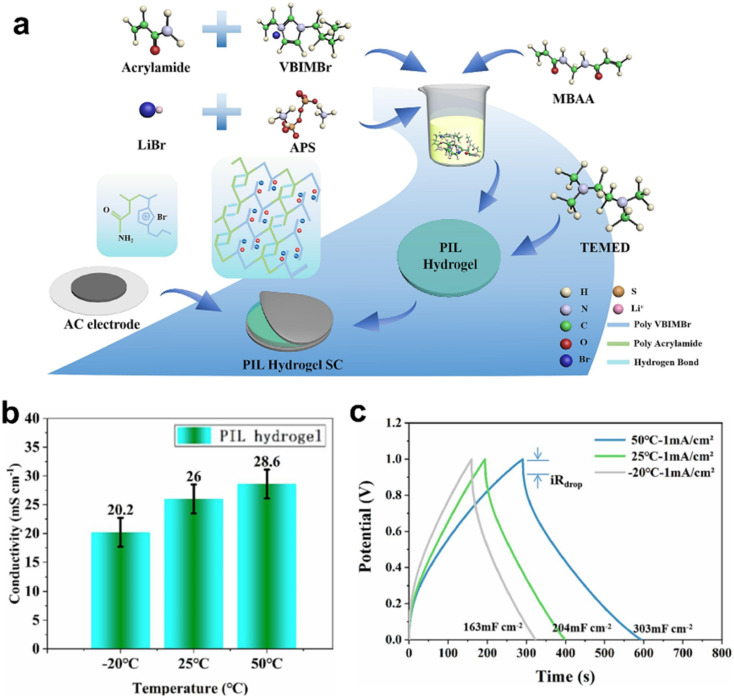
Anti-freezing PIL hydrogel electrolytes enabled by engineering hydrogen-bond networks. (a) Schematic diagram of the synthesis process of the PIL hydrogel. (b) Temperature-dependent ionic conductivity of the PIL hydrogel. (c) GCD profiles of PIL hydrogel-based supercapacitors at various temperatures with a current density of 1 mA cm^−2^. Reproduced with permission.^50^ Copyright 2023, Elsevier.

Compared to traditional hydrogels, ionic liquid gels exhibit superior conductivity at low temperatures and improved thermal stability. Poly(ionic liquid) (PIL) hydrogels, synthesized from ionic liquid monomers, integrate the unique properties of ionic liquids with the structural advantages of polymer scaffolds. This enables PIL hydrogels not only to retain the physical/chemical characteristics of ionic liquids but also to benefit from the mechanical strength of polymers. For example, He *et al.* developed an innovative anti-freezing electrolyte based on a PIL hydrogel, which demonstrates high conductivity for energy storage applications. The anti-freezing gel electrolyte shows an impressive conductivity of 26 mS cm^−1^, while the assembled supercapacitors exhibit outstanding electrochemical performance, with a specific capacitance of 204 mF cm^−2^ at 1 mA cm^−2^. Notably, the supercapacitors retain 79.9% of their capacitance even at −20 °C, highlighting their remarkable low-temperature stability (Fig. 12).^50^

## 4. Theoretical simulation and advanced characterization

With rapid advancement of computational modeling methods and advanced characterization techniques, we can efficiently screen low-temperature electrolyte systems, better understand experimentally observed phenomena, and explore reaction mechanisms at low temperatures.^69–71^ For instance, the solvation/desolvation processes are the critical factors influencing the electrochemical performance at low temperatures. Therefore, understanding and decoupling the solvation structure is key to regulating these processes and optimizing electrolytes. Theoretical simulations and advanced characterization are powerful tools for achieving this. Specifically, theoretical calculations can be employed to predict the solvation structure, ion transport kinetics and thermodynamic stability. Meanwhile, advanced characterization technologies, such as infrared (IR) spectroscopy, Raman spectroscopy, and nuclear magnetic resonance (NMR) technology, offer valuable insights into the solvation/desolvation process and ion transport behaviors by identifying characteristic peaks associated with the components in the electrolyte from an experimental perspective. Thus, mutual verification between theoretical calculations and spectroscopic characterization could be achieved.

Recently, theoretical simulations have become essential tools in low-temperature electrolyte research.^72^ Density functional theory (DFT), molecular dynamics (MD) and machine learning (ML) methods offer a deeper understanding of atomic or molecular-level interactions, providing valuable information such as ion coordination numbers, binding energies, electrostatic potentials, and the entropy of electrolytes.^73,74^ These methods provide valuable insights into electrolyte behavior at extremely low-temperatures that are otherwise difficult to access, such as electronic structure effects, solvation structure, and ionic diffusion mechanisms. The target temperature of multiscale models for electrolyte systems can be easily adjusted by modifying the code. To be more precise, DFT calculations can be utilized to elucidate the electronic-level interactions among electrolyte components, enabling a quantitative understanding of solvent–solvent, ion–solvent, and cation–anion interactions. Such first-principles approaches provide insights into binding energies, charge distributions, and interaction strengths at the atomic scale.^47^ Besides, MD simulations are typically employed to capture the time-resolved, statistical behavior of ions and solvent molecules, offering detailed information on solvation structures, dynamic rearrangements, and ion transport mechanisms under various conditions.^75^ Moreover, ML methods are being increasingly employed to expedite the screening of advanced low-temperature electrolytes, thereby circumventing the time- and resource-intensive trial-and-error approach. ML enables systematic analysis of experimental and theoretical data to establish electrolyte structure–property relationships, facilitating efficient identification of promising candidates. As validated through high-throughput experimentation, these findings can be incorporated into databases to further refine ML models. This approach not only accelerates the discovery of high-performance low-temperature electrolytes but also enables accurate prediction of electrochemical properties, demonstrating significant potential for advancing low-temperature energy storage systems.^76^ Such theoretical methods provide valuable insights into charging mechanisms when combined with advanced characterization techniques.^77–79^

**Fig. 13:**
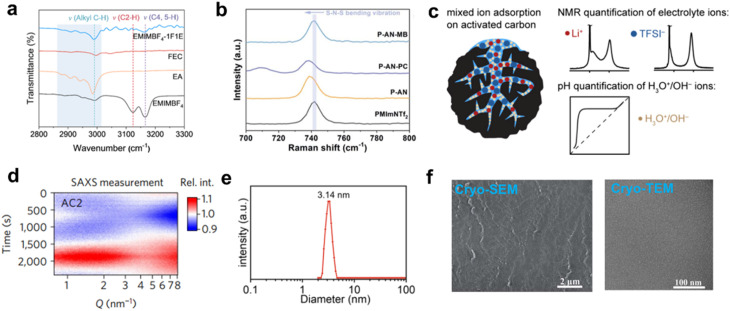
Overview of advanced characterization techniques for low-temperature electrolytes: (a) IR spectroscopy, reproduced with permission.^81^ Copyright 2024, Elsevier. (b) Raman spectroscopy, reproduced with permission.^47^ Copyright 2022, Royal Society of Chemistry. (c) NMR spectroscopy, reproduced with permission.^87^ Copyright 2024, American Chemical Society. (d) SAXS analysis, reproduced with permission.^79^ Copyright 2017, Springer Nature. (e) DLS measurements, reproduced with permission.^90^ Copyright 2023, Wiley-VCH. and (f) Cryo-EM imaging, reproduced with permission.^61^ Copyright 2022, Royal Society of Chemistry.

As for advanced characterization techniques, IR spectroscopy plays a key role in the study of low-temperature electrolytes by providing valuable information about molecular vibrations and interactions.^80^ For the analysis of samples at low temperatures, special sample holders (such as liquid nitrogen-cooled sample cells) are typically used to keep the samples stable at such extreme temperatures. In general, IR spectroscopy serves as a useful tool for analyzing the solvation structure, offering valuable insights into the interactions between the solvent, additives, and ions in the electrolyte. For instance, Jiang *et al.* used IR spectroscopy to study the solvation structure of the electrolyte. With the addition of fluoroethylene carbonate (FEC)/ethyl acetate (EA), the alkyl C–H peak was found to be blue-shifted, suggesting an altered environment surrounding the C–H group on the imidazolium cation within the EMIMBF_4_-FEC/EA electrolyte compared to pure EMIMBF_4_. Furthermore, the addition of EA and FEC induced noticeable shifts of 2 and 3 cm^−1^ in the vibrational frequencies *v*(C2–H) and *v*(C4,5–H) of the imidazolium ring. Such significant shifts imply enhanced hydrogen bonding at these positions, which promotes a more uniform dispersion of the ionic liquids. This effect contributes to the decreased viscosity and enhanced ionic conductivity of the EMIMBF_4_-1F1E electrolyte over a broad temperature range (Fig. 13a).^81^ Moreover, the interactions can be notably altered at low temperatures due to changes in molecular motion and the formation of ion–solvent complexes. IR tests at low temperatures allow for the study of how the vibrational modes of electrolyte components change with temperature. Additionally, low temperatures may induce crystallization or phase transitions in certain electrolyte components, and such structural transformations can also be detected and characterized by IR spectroscopy. In short, IR spectroscopy can help researchers gain a deeper understanding of electrolyte properties and design advanced electrolytes for supercapacitors.

Complementary to IR spectroscopy, Raman spectroscopy is a powerful technique that offers insights into molecular vibrations, bond interactions, and the local environment of ions and solvents, making it especially useful for studying electrolyte.^82^ Raman spectroscopy can identify specific functional groups in electrolyte components (such as solvents, ions, and additives) and monitor shifts in Raman peaks to detect changes in the chemical environment as temperature decreases. This helps track slight structural changes that affect electrolyte performance at low temperatures. For instance, Raman spectroscopy was employed to investigate the structural modulation of ionic liquid-based electrolytes induced by organic co-solvents. The characteristic S–N–S bending vibration of the TFSI^−^ anion, observed at 740–750 cm^−1^, exhibited a red shift from 741.9 cm^−1^ to 738.5 cm^−1^ in the P-AN and P-AN-PC systems (P: [ionic liquid], AN: ACN, PC: methyl butyrate), indicative of hydrogen bonding between TFSI^−^ and co-solvents. In contrast, no spectral shift occurred in the presence of ACN and methyl butyrate (MB), suggesting preferential hydrogen-bond interactions of these solvents with the ionic liquid's cation rather than the TFSI^−^ anion. This coordination likely weakens the cation–anion interaction, facilitating the improved dispersion of ionic liquid. Furthermore, these interactions can account for the significant reduction in viscosity and the enhanced conductivity observed in the P-AN-MB system at low temperatures (Fig. 13b).^47^

NMR spectroscopy can provide detailed information about the local environment of solvents, ions, and their interactions with the electrolyte.^78,83–86^ In particular, NMR can be utilized to probe the interactions between different components of the electrolyte. By analyzing changes in chemical shifts and relaxation times, NMR provides insights into the local structural changes of the electrolyte, helping to identify the most suitable electrolyte formulations for supercapacitors. Representatively, NMR spectroscopy was employed to investigate the absorption behavior of an aqueous lithium bis(trifluoromethanesulfonyl)imide (LiTFSI) electrolyte by commercial activated carbon. The NMR results show rapid ion exchange between the bulk electrolyte and the ions within the carbon pores. These fast exchange processes significantly influence the apparent concentration of adsorbed Li^+^ and TFSI^−^ ions, as quantified by NMR. The experiment suggests that as the electrolyte becomes more acidic, the carbon pores exhibit increased ionophilicity, which has crucial effects on the charge storage mechanisms in electrochemical systems (Fig. 13c).^87^ NMR instruments equipped with cryoprobes can perform the tests at low temperatures, revealing how ions are solvated in different electrolyte composition at low temperatures, which is critical for optimizing low-temperature performance of supercapacitors. Examining changes in the NMR spectra at various temperatures is important for understanding how the charge storage capability is affected by temperature.

Small-angle X-ray scattering (SAXS) can provide valuable insights into the ion adsorption behaviors in nano and sub-nano channels.^71,88,89^ As a typical example, Prehal *et al.* elucidated voltage-dependent ion redistribution in aqueous electrolytes confined within three microporous carbons using *in situ* SAXS. By coupling experimental SAXS data with theoretical simulations, their methodology reveals the correlations between electric-field-driven ion confinement/desolvation and capacitive behavior, advancing the rational design of carbon-based energy storage systems (Fig. 13d).^79^ In general, electrolyte ions may exhibit different behaviors at low temperatures, such as increased ion pairing or aggregation due to lower thermal motion. SAXS can detect changes in the scattering pattern that correspond to these structural changes. By analyzing the scattering profile, researchers can gain insights into the solvation structure and how they are influenced by temperature or electrolyte composition.

Dynamic light scattering (DLS) is a versatile technique commonly used to analyze particle size distribution and dynamics in colloidal suspensions, making it particularly useful for studying the behavior of electrolytes at low temperatures.^90^ DLS can be used to monitor temperature-dependent changes in the size of ion clusters, aggregates, or nanoparticles in low-temperature electrolytes. In liquid electrolytes, particularly ionic liquids or gel-based systems, ions may aggregate or form clusters at lower temperatures, potentially affecting conductivity and electrochemical performance. DLS allows for the detection and quantification of such aggregation, providing insights into its impact on ionic transport. If aggregation results in larger clusters or phase separation, DLS can help inform adjustments to electrolyte composition to minimize negative effects on supercapacitor performance. For instance, Shi *et al.* employed DLS measurements to unravel micelle self-assembly behavior in a 1 M LiFSI electrolyte, revealing particles with an average diameter of approximately 3.14 nm. The observed Tyndall effect further supports these findings. Both simulation and experimental data confirm that the introduction of the amphiphilic 1,1,2,2-tetrafluoro-3-methoxypropane (TFMP) cosolvent facilitates the self-assembly of the electrolyte, leading to the formation of a core-shell-like solvation structure. This distinctive solvation structure enhances ion conductivity and enables facile desolvation, demonstrating superior cyclability at low temperatures (Fig. 13e).^90^

Cryogenic electron microscopy (Cryo-EM) is a cutting-edge imaging technique primarily used for the detailed study of biological macromolecules at high resolution, but it has also found applications in the study of electrolytes.^91–94^ Cryo-EM provides significant advantages in examining the structural and morphological properties of electrolytes at near-freezing temperatures. At low temperatures, electrolytes may undergo phase transitions like crystallization, gel formation, or aggregation, which can hinder ion mobility and decrease conductivity. Cryo-EM allows for high-resolution imaging of these processes, offering valuable insights into electrolyte behavior under low-temperature conditions and their impact on supercapacitors performance. Real-time imaging can identify these transitions, aiding the development of more stable electrolytes for supercapacitors in cold environments. For example, Tu *et al.* used cryo-SEM and cryo-TEM to examine the morphological characteristics of the ion-conducting gel (IG). SEM observations of the IG membrane exhibit a smooth, homogeneous surface without visible crystalline CuSO_4_ aggregates, suggesting favorable compatibility between the PVA matrix and the CuSO_4_ species. Furthermore, cryo-TEM analysis was conducted to explore the internal structure of the IGs. The resulting images revealed an amorphous morphology within the bulk phase, with no distinct signs of CuSO_4_ crystallization. These findings imply that the PVA solution underwent freezing into a glassy, non-crystalline state, in which Cu^2+^ and SO_4_^2−^ ions were uniformly dispersed. The uniform distribution of molecules/ions will facilitate the formation of ion channels, ensuring efficient ion migration within the IGs (Fig. 13f).^61^

## 5. Summary and perspectives

**Fig. 14:**
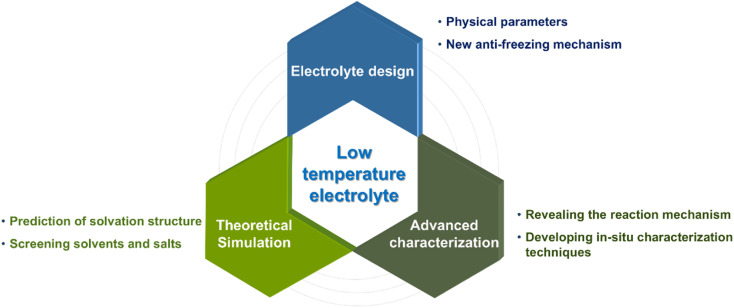
Overview and future perspectives on the development of low-temperature electrolytes for high-performance supercapacitors.

Supercapacitors are ideal for applications requiring rapid energy delivery and long cycle life, particularly in hybrid energy systems where power needs to be delivered quickly or intermittently. However, they suffer from significant capacitance degradation or even failure at low temperatures. Therefore, the development of advanced electrolyte systems is essential for achieving high-performance supercapacitors under low-temperature conditions. In this perspective, we first analyze the fundamental limitations impairing electrolyte performance at subzero temperatures and then evaluate state-of-the-art design strategies for low-temperature electrolytes. We further present a systematic summary of theoretical simulations and advanced characterization techniques to accelerate electrolyte optimization, which can help researchers in screening optimal solvents and salts, monitoring solvation/desolvation behaviors, and unveiling reaction mechanisms (Fig. 14). Despite notable advancements in the development of low-temperature electrolytes, several critical challenges persist that hinder their widespread application. Future research endeavors should concentrate on the following key aspects:

(1) Optimizing the multiple physical parameters of electrolytes to achieve optimal overall performance and establishing standardized evaluation criteria: recent studies have predominantly focused on pursuing extremely low operational temperatures, with excessive emphasis placed on depressing the freezing point of electrolytes. However, other critical performance parameters of electrolytes are often neglected, including viscosity, ionic conductivity, and electrochemical stability window. Such unilateral optimization approaches may compromise the overall electrochemical performance under low-temperature conditions. Therefore, a more comprehensive set of evaluation criteria for low-temperature electrochemical performance should be established to enable a more objective and holistic assessment of device behavior at low temperatures. Future research should embrace a holistic design paradigm that concurrently optimizes multiple physicochemical parameters alongside the development of low-melting-point electrolytes, thereby enabling a comprehensive enhancement in low-temperature electrochemical performance.

(2) Developing novel strategies to construct high-performance electrolyte systems: beyond conventional approaches such as salt selection or solvent optimization, future research should explore unconventional electrolyte architectures. For instance, integrating entropy-driven design principles, weak solvation strategies, deep eutectic solvents, and advanced additive engineering can enable the simultaneous optimization of multiple critical parameters of electrolytes and improve low-temperature performance. In the future, collaborative efforts spanning materials, chemistry, and physics science are anticipated to unveil novel anti-freezing mechanisms and foster innovative breakthroughs in the design of next-generation electrolyte systems, thereby achieving robust electrochemical performance under extreme temperature conditions.

(3) Investigating and establishing the connection between the microstructure and the low-temperature performance of electrolyte: for instance, the solvation structure and desolvation process play a critical role in governing ion transport at the solid–liquid interface. At low temperatures, sluggish ion mobility and hindered interfacial charge transfer often arise from rigid solvation shells and limited desolvation kinetics. A detailed understanding of how the electrolyte's local coordination environment, ion pairing behavior, and solvent dynamics evolve under low-temperature conditions can provide critical insights into the ion transport limitations. Moving forward, tailoring solvation structures through molecular design, such as the incorporation of asymmetric solvents, weakly coordinating solvents, or fluorinated species, holds great promise in promoting efficient desolvation and enhancing ion transport kinetics. Ultimately, such microstructure-performance insights will lay a solid foundation for the rational design of next-generation electrolytes specifically engineered for high-performance supercapacitors in low-temperature environments.

(4) Developing advanced computational methods to screen effective electrolyte components and predict their low-temperature performance: although DFT calculations and MD methods can shed new light on the microscopic structure of electrolytes, the rise of ML technology will provide more intelligent solutions for the design of low-temperature electrolytes. By training predictive models on high-quality experimental and simulation datasets, ML methods can rapidly identify correlations between molecular structure, solvation characteristics, and low-temperature electrochemical performance. Furthermore, ML methods offer powerful capabilities in identifying latent patterns and complex nonlinear relationships that often elude conventional analytical approaches. The advancement of ML methods can effectively reduce the cost of trial-and-error processes and improve efficiency. In the long term, the construction of comprehensive electrolyte databases and the adoption of active learning and generative models may enable the inverse design of low-temperature electrolytes with desired physicochemical properties.

(5) Developing advanced *in situ* characterization techniques to unveil energy storage mechanisms at low temperature: currently, many existing characterization techniques lack low-temperature operability. This presents a significant bottleneck in the mechanistic understanding of low-temperature electrochemical phenomena. Developing novel cell designs or equipment that can adapt current characterization tools to low-temperature environments could provide new insights into this area. For instance, innovative efforts are needed to engineer bespoke electrochemical cells and integrated setups capable of maintaining precise temperature control while ensuring compatibility with a wide range of spectroscopic and microscopic techniques. The development of cryo-compatible *in situ* cells, vacuum-insulated chambers, or microfluidic platforms with localized temperature regulation could enable real-time monitoring of solvation dynamics, ion transport behavior, and electrode–electrolyte interface evolution under realistic working conditions. By enabling direct observation of the structural, chemical, and electrochemical changes that occur in low-temperature environments, these advanced characterization strategies will provide critical insights for the rational design of low-temperature electrolyte systems for supercapacitors.

(6) Enhancing low-temperature performance by optimizing supercapacitor components beyond the electrolyte: in addition to the electrolyte, the construction of advanced electrode materials tailored for low-temperature electrolytes is of paramount importance. While existing studies predominantly emphasize lowering the freezing point and improving the ionic conductivity of low-temperature electrolyte, insufficient attention has been paid to the design and optimization of electrode materials that are well-matched with such electrolytes. Given their pivotal role in supercapacitors, the microstructure, specific surface area, pore architecture, and electrical conductivity of electrode materials critically affect device performance. Thus, it is imperative to advance electrode materials that are compatible and synergistic with the electrolyte systems. Moreover, other components such as current collectors, separators, conductive additives, and binders also exert significant influence on the electrochemical performance at low temperatures. Therefore, it is essential to rationally optimize and integrate these components to achieve optimal electrochemical performance of supercapacitors at low temperatures.

Overall, despite significant advancements in low-temperature supercapacitors in recent years, the design of efficient electrolytes suitable for low-temperature applications remains a critical challenge. Considering the rising demand for energy storage systems capable of reliable operation in cold environments, the prospects for advanced low-temperature electrolytes are highly promising. With intensified research efforts in this field, novel computational approaches and advanced characterization techniques are being continuously developed to elucidate the microstructure of electrolytes and investigate their energy storage mechanisms. We anticipate continuous breakthroughs that will ultimately accelerate the commercialization and widespread applications of low-temperature supercapacitors.

## Author contributions

C. M. was the main contributor to the writing of the manuscript, whereas Z. W. was the main contributor to its revision. All authors participated in discussions and revisions.

## Conflicts of interest

The authors declare no conflicts of interest.

## Data Availability

No primary research results, software or code have been included and no new data were generated or analysed as part of this review.
